# Inactivation of Individual SeqA Binding Sites of the *E*. *coli* Origin Reveals Robustness of Replication Initiation Synchrony

**DOI:** 10.1371/journal.pone.0166722

**Published:** 2016-12-08

**Authors:** Jyoti K. Jha, Dhruba K. Chattoraj

**Affiliations:** Laboratory of Biochemistry and Molecular Biology, Center for Cancer Research, National Cancer Institute, National Institutes of Health, Bethesda, MD, United States of America; University of Massachusetts Medical School, UNITED STATES

## Abstract

The *Escherichia coli* origin of replication, *oriC*, comprises mostly binding sites of two proteins: DnaA, a positive regulator, and SeqA, a negative regulator. SeqA, although not essential, is required for timely initiation, and during rapid growth, synchronous initiation from multiple origins. Unlike DnaA, details of SeqA binding to *oriC* are limited. Here we have determined that SeqA binds to all its sites tested (9/11) and with variable efficiency. Titration of DnaA alters SeqA binding to two sites, both of which have overlapping DnaA sites. The altered SeqA binding, however, does not affect initiation synchrony. Synchrony is also unaffected when individual SeqA sites are mutated. An apparent exception was one mutant where the mutation also changed an overlapping DnaA site. In this mutant, the observed asynchrony could be from altered DnaA binding, as selectively mutating this SeqA site did not cause asynchrony. These results reveal robust initiation synchrony against alterations of individual SeqA binding sites. The redundancy apparently ensures SeqA function in controlling replication in *E*. *coli*.

## Introduction

Chromosomal replication initiation in *Escherichia coli* is controlled by a combination of positive and negative regulators [1, 2]. The primary positive regulator is the initiator protein DnaA [3, 4]. A negative regulator, SeqA, was identified years later although an obligatory requirement for such a regulator was predicted earlier [1, 5, 6]. How the different regulators function in allowing replication once per cell cycle and at a particular time of the cell cycle is not fully understood [7].

**Fig 1:**
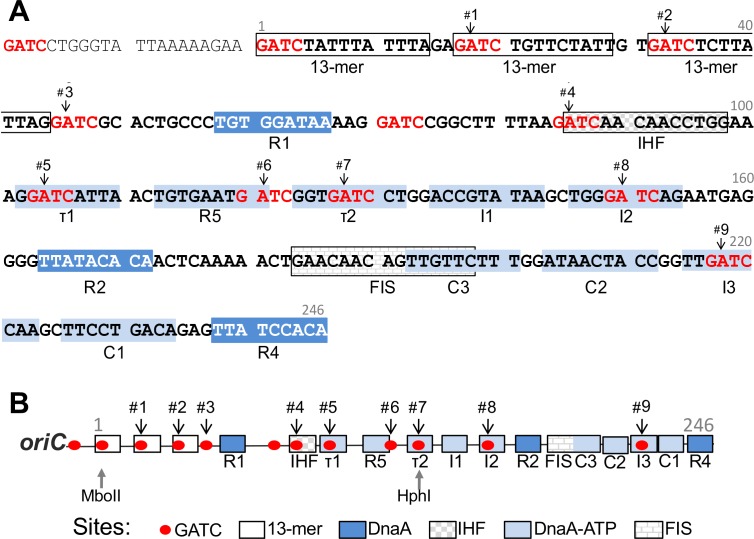
Sequence of *oriC* showing its major protein binding sites. (A) Sequence of *oriC* that includes the minimal region (coordinates 1–246) required for origin function. The coordinate 1 correspondence to 3923744 of gb|U00096.3|. The region includes several GATC sites (shown in red) which are methylated by the Dam methylase enzyme. There are three 13-mer repeats of AT rich sequences where the origin initially unwinds. The remainder of the origin has mainly 9-mer DnaA binding sites as well as sites for binding IHF and FIS proteins. DnaA sites have either high (R1, R2 and R4) or low (τ1, R5, τ2, I1, I2, C3, C2, I3 and C1) affinity for DnaA. The numbers #1–9 mark the GATC sites studied here. A TaqI site (TCGA) overlapping each of the nine GATC sites was created by converting their two upstream bases to TC (NNGATC to TCGATC). (B) A linear map of *oriC* features described above. The map also shows location of sites for restriction enzymes MboII and HphI that naturally occurs in *oriC*.

Replication regulators in *E*. *coli*, including SeqA, modulate mainly the interaction of the initiator DnaA with the origin of replication, *oriC* [1, 8]. Binding sites of DnaA and SeqA are distributed throughout the chromosome, the density of both the sites being highest at *oriC* (11 sites of each within the 246 bp *oriC*) [9]. The affinity of DnaA for its binding sites in *oriC* varies, partly due to considerable variation of the site sequences from the 9-mer consensus sequence TTATC/ACACA [10]. The high affinity sites, whose sequences match the consensus sequence, are R1, R2 and R4 (Fig 1A). DnaA complexed with ATP or ADP binds equally well to these sites, and remains bound throughout the cell cycle [8]. In contrast, most of the low affinity sites such as τ1, R5, τ2, I1, I2, C3, C2, I3 and C1 show a preference for binding DnaA-ATP, the form that is required for initiation [9]. An exception is R5, which is a low affinity site but binds DnaA-ATP and DnaA-ADP equivalently, implying that low affinity and preference for DnaA-ATP are two different attributes [11]. Maximal binding to the weak sites occurs at the time of initiation when the level of DnaA-ATP peaks in the cell cycle [12]. Regulation of binding to these low affinity sites is the key regulatory strategy for controlling initiation in *E*. *coli*.

DnaA-ATP is also required for binding to *oriC* in the region containing the three 13-mers [13] (Fig 1A). The strands of *oriC* initially open in this region allowing helicase loading. DnaA binds to one specific strand of the open region, which helps to stabilize the open state and facilitate helicase loading to the other stand [14–16].

The characteristics of SeqA binding to DNA are distinct from those of DnaA. SeqA binds to GATC sites, which are targets for adenine methylation by the deoxyadenosyl methyltransferase (Dam) enzyme. SeqA prefers to bind to hemimethylated (HM) GATC sites that are generated from fully methylated sites upon replication and SeqA binding prolongs the HM state [1]. The prolongation is particularly long (~1/3^rd^ cell generation) at two loci, *oriC* and the promoter of the *dnaA* gene that also has a high density of GATC sites. Several of the low affinity DnaA binding sites of *oriC* have overlapping GATC sequences and DnaA binding to R5, I2 and I3 sites reduces in the presence of SeqA [8]. SeqA thus appears to delay initiation by interfering with DnaA binding to *oriC*.

During rapid growth, *E*. *coli* initiates DNA replication on a partially duplicated chromosome, causing overlapping rounds of replication and the presence of multiple origins in duplicating chromosomes. Nonetheless, replication initiates from all the origins within a narrow window of time and initiates from each origin only once per cycle (initiation synchrony) [17]. This requires the presence of SeqA protein, and in *seqA* mutants replication becomes highly asynchronous [1]. SeqA binding to newly generated HM origins that helps to prevent their additional firing in the same cell cycle is called sequestration. The sequestration of newly replicated origins also prevents them from competing with the yet-to-be replicated origins and thereby helps in their firing [18].

SeqA could inhibit initiation in additional ways: 1. By prolonging the HM state, which compromises *oriC* activity since the origin fires more efficiently when it is fully methylated [19]. 2. By sequestering the DnaA promoter, which reduces initiator synthesis following replication initiation and this helps to reduce premature reinitiation [20]. 3. By binding to fully methylated DNA, specifically to the left half of *oriC in vitro* [21, 22]. This is believed to inhibit initiation by interfering with origin opening 4. By absorbing negative supercoils, which are required for initiation [23]. This activity of SeqA does not require DNA to be methylated, a finding that is consistent with the fact that SeqA overproduction is inhibitory in *dam* mutant cells [1]. Thus, although primarily known as a HM DNA binding protein, SeqA can bind to fully methylated DNA and can also bind non-specifically to unmethylated DNA.

SeqA binding to *oriC* thus appears as complex as that of DnaA, and the requirements of SeqA binding in negative regulation of replication remain to be fully defined. Here we have focused on SeqA binding related to initiation synchrony. We show that 1) SeqA binds to all the GATC sites of *oriC* that we tested (9/11) and the extent of binding varies among the sites; 2) SeqA binding is altered to two of GATC sites upon titration of DnaA but this does not affect initiation synchrony; and 3) Initiation synchrony is also not affected by mutations in individual GATC sites. These results suggest that initiation synchrony is a robust process that does not depend on varied SeqA binding across *oriC* and integrity of any of the single GATC sites of *oriC*.

## Materials and Methods

### Bacterial strains, plasmids, primers and media

Bacterial strains, plasmids and primers used in this study are listed in Tables A-C in S1 File. *E*. *coli* was grown in either LB, or 1X M63 medium (KD Medical) supplemented with 0.005% thiamine, 0.2% glucose and additionally 0.1% or 0.5% casamino acids (CAA) depending on the experiment. Antibiotics were used at the following concentrations: ampicillin, 100 μg/ml; tetracycline, 12.5 μg/ml; chloramphenicol, 25 μg/ml and zeocin, 20 μg/ml.

### Construction of *E*. *coli* mutants with mutated *oriC*

WT *oriC* (coordinates 3925715–3926016 of K12-MG1655 strain gb|U00096.3|) was amplified from *E*. *coli* genomic DNA from strain MG1655 (BR1703) by using primer pairs jj15+ jj16. The PCR product was digested with EcoRI and BamHI, and ligated to similarly digested pEM7-Zeo vector, resulting in plasmid pJJ04. Bases were substituted within *oriC* to create TaqI sites or alter GATC sites by site-directed mutagenesis (using Hifi-KAPA hot start ready mix PCR kit, KAPA-Biosystems), using pJJ04 as template and primers. The plasmids with new TaqI sites are pJJ06,-40,-41,-46,-47,-48,-49,-55 and -07, and with altered GATC sites are pJJ-385,-386,-387,-388,-389,-379,-391,-369,-370,-371,-372,-374,-377,-390,-415 and -416. Note that we created TaqI sites overlapping 9/11 GATC sites (Fig 1*A*). The GATC in the left 13-mer was probed earlier and here using MboII. Additionally, the GATC site next to R1 (called 3*) was probed in some of the experiments.

### Transfer of *oriC* mutations from plasmid to chromosome by recombineering in *E*. *coli*

*oriC* (3925715–3926016) was amplified by PCR using pJJ04 or its mutant derivatives as templates and jj21+jj35 as primers. The 5’ and 3’ ends of these primers have homology with chromosomal flanks of *oriC* (*zeo-mioC* and *gidA*). The linear PCR products containing the WT or mutated *oriC* and the linked *zeo* cassette were transferred to the chromosome of the *E*. *coli* strain BR1703 (WT) or BR1704 (Δ*seqA*) by the lambda red recombineering method [24]. The strains were first made competent for recombineering by introducing the mini-λ tet plasmid carrying the Red recombination genes. The resulting strains CVC2073 and CVC2092 were used to introduce the linear PCR products by electroporation. After mutation transfer, the strains were cured of the mini-λ tet plasmid, and the sequence of the mutated origins was confirmed by PCR; the origin region (3925517–3926140) was amplified using primers jj40+jj42 and the amplified product was sequenced using the primer jj122 (3925620–3925642).

In order to delete the *zeo* cassette from the *oriC*-*zeo*#1–9 strains, they were transformed with pSIM5 plasmid. A PCR product containing *FRT*-Kan-*FRT* and flanks of ~40 bp with homology to the flanks of the *zeo* cassette was generated using pRFB105 plamid as template and jj184+jj185 as primers. This PCR product was electroporated into *oriC*-*zeo*#1–9 strains, and the transformants were screened for Kan^R^ and Zeo^S^. The Kan replacement was confirmed by PCR with Kan flanking primers jj40+jj42. The Kan marker was excised using pCP20 that left one *FRT* site (35 bp). The final *oriC-FRT*#1–9 strains with a single *FRT* scar were confirmed by PCR amplification using primers jj40+jj42 and sequencing the PCR products with jj122.

The *oriC*-FRT#1–9 strains were individually made *dam* minus by P1 transduction. The phage stock was derived from *dam*::Tn*9* Cm^R^ strain, BR2786. The loss of *dam* was confirmed by flow cytometry.

### Flow cytometry

Cultures of *E*. *coli* were grown in supplemented 1X M63 medium as described above. The cultures were grown to OD600 ~ 0.15 and processed for flow cytometry before and after replication run-out in the presence of rifampicin (100 μg/ml) and cephalexin (10 μg/ml) for four hours as described [25]. The peak fluorescence intensity of an overnight grown *E*. *coli* culture of BR1703 in M63 + 0.2% glycerol medium (without casamino acids) was taken to represent one full chromosome. From the flow cytometry profiles that represent multimodal distribution of cells, each peak of which represent cells with a fixed number of chromosomes, origins/cell was calculated from the product of the chromosome number and the fractional area of the total distribution representing that number of chromosomes, and summing the products from all the peaks of the distribution. The asynchrony index was calculated by measuring the area of the distribution containing three chromosomes compared to total area containing all the chrmosomes (two, three- and four chromosomes under the present experimental set up) [26]. Fraction of uninitiated cells is the present study is the fractional area of the distribution representing two-chromosome cells. This was used to measure initiation mass. 100,000 cells were analyzed in each panel using the Flow Cytometer BD LSR Forestessa SORP II (BD Biosciences). The triggering volt was set at 200 for the side scatter.

### Southern blotting

**Fig 2:**
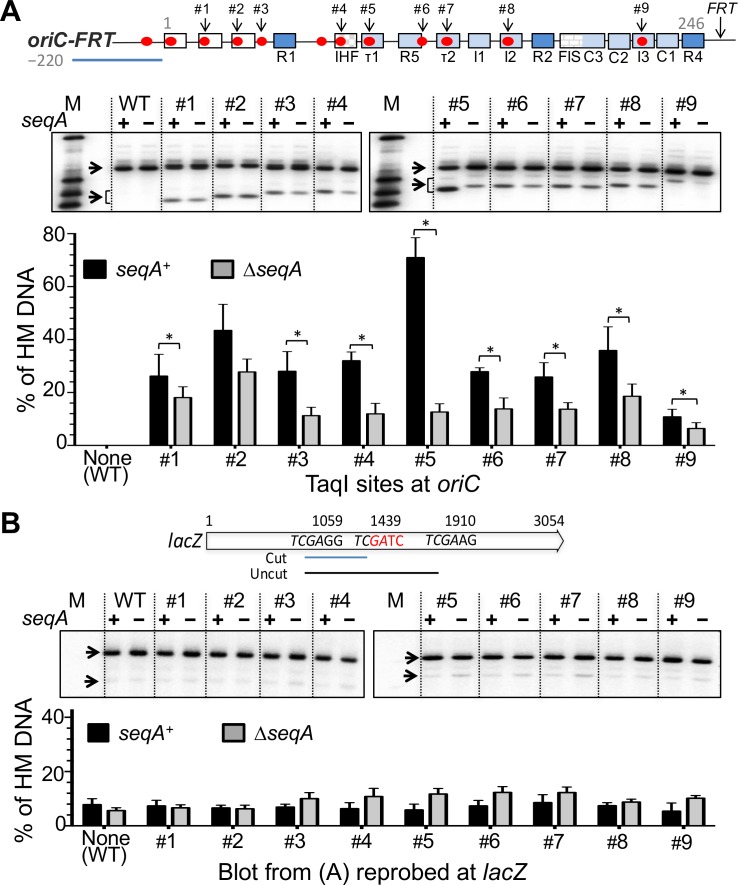
Quantification of hemimethylated DNA level at different GATC sites of *oriC* in *seqA*^+^ and Δ*seqA* strains. (A) The top line shows a schematic map of *oriC* as in Fig 1B, which also includes the location of an *FRT* site that was present in all the strains used in this figure. The autorad is a representative Southern blot of genomic DNA from *seqA*+ and Δ*seqA* strains after digestion with TaqI. The blots were probed with a 220 bp PCR amplified fragment (uisng primers jj40+jj41) homologuous to the left flank of *oriC* (blue line). M represents *oriC* specific markers (1100bp, 460 bp, 330 bp and 240 bp). The arrows indicate the bands in which the test GATC sites were either fully methylated, thus resistant to TaqI digestion (upper bands), or HM and sensitive to TaqI digestion 50% of the time (lower bands), as explained in Figure A(A) in S1 File. The intensity of the lower bands was multiplied by a factor of two to calculate the relative level of HM DNA (lower panel). [Note that the separation of the two bands is gradually narrowing because of shifting position of TaqI site within *oriC* with respect to the two TaqI sites that flank *oriC* (see also Figure A(B) in S1 File). The upper fragment is generated when TaqI digests the two *oriC* flanking sites but not the TaqI site within *oriC*.] The black and gray bars represent mean HM DNA levels in *seqA*^+^ and Δ*seqA* strains, respectively, determined from three cultures innoculated with independent colonies (biological replicates). The error bar represents one standard deviation of the mean. Asterisks indicate pairwise comparisons that were statistically distinguishable (*P* < 0.05, students *t-*test). (B) Same as (A) except that the *oriC* probe was stripped off the blot, which was then reprobed with a PCR product (using primers jj193+jj194) from a region in *lacZ* (blue bar) [28]. The error bars represent variability in repeated measurements of the same blot (technical replicates).

Genomic DNA was isolated from LB grown log phase cultures (OD600 ~0.3) using the Genelute Bacterial Genomic DNA kit (Sigma). The DNA was digested for an hour with 3 units of MboII or HphI at 37°C, or TaqI at 65°C (New England Biolabs). Partial digestion of genomic DNA from *dam* minus strains was performed with 3 units of TaqI at 55°C for 10 min. The digested products were resolved in a 1.3% agarose gel. An *oriC* region (3925517–3925542) was PCR amplified using primers jj40+jj41 and the product was used as the probe. The probe for the HphI digested blot covered the region 3925275–3925633 and was amplified using primers jj168+ jj169. The probe for the *oriC* external-marker in *lacZ* covered the region 364871–365085 and was amplified using primers jj193+jj194. The probes were made radioactive using the Redi-PrimeII random primer labeling kit (GE Healthcare) and [α-32P] dCTP (Perkin Elmer). The band intensities were recorded and quantified as described earlier [27]. The blots were re-probed for an external marker (in *lacZ*) located ~365 kb away from *oriC*. As reported earlier, the level of HM DNA at *lacZ* was significantly lower compared to the levels at TaqI sites created in *oriC* (Fig 2B).

## Results

### Introduction of new probing sites for SeqA binding across *oriC*

Semi-conservative replication of fully methylated *oriC* generates HM sister origins. The HM state is prolonged upon SeqA binding and this prolongation has been used as a proxy for SeqA binding *in vivo* [1, 28]. The detection of SeqA binding required the ingenious approach of utilising restriction enzymes whose sites overlap the GA of GATC sites and whose activities are inhibited when the adenine residue of GATC is methylated. However, for one of the two HM sister sites, the methylated adenine of the template strand falls outside of the restriction site, rendering the site sensitive to restriction digestion (Figure A(A) in S1 File). If HM state is prolonged at any site, a greater proportion of digested DNA is recovered from that site.

Restriction sites overlapping GATC and suitable for probing the HM state are available at only two of the 11 GATC sites in *oriC*: one overlaps with a MboII site and the other with a HphI site (Fig 1B). To probe the GATC sites across the entire *oriC*, we introduced GATC overlapping sites for an adenine methylation-sensitive enzyme, TaqI. TaqI was chosen because creating its site, TCGA, required making at most two changes to *oriC*. Note that changes outside of GATC are not known to affect SeqA binding [29–31]. We mutated one or two upstream bases of GATC to TC to create TCGATC sequences overlapping nine of the GATC sites of *oriC* (#1 - #9, Fig 1A and 1B; Figure A(B) in S1 File). The TaqI sites were initially created in a *oriC* plasmid, in which *oriC* was linked to a drug marker, *zeo*. The mutated origins with a TaqI site were designated *oriC*-*zeo*#1–9. We also deleted the *zeo* marker and replaced it with an *FRT* site (35bp). This set of mutants was designated *oriC-FRT*#1-9.

We tested the *FRT* set of mutants for initiation efficiency (origin/cell mass) and initiation synchrony by replication run-out using flow cytometry [17]. The origin/cell-mass of the mutants was 0.97 ± 0.05 compared to 1 for the WT, and the asynchrony index 7.8 ± 4.0% compared to 3.9% for the WT (Figure B in S1 File; Table 1). The latter result indicates that the mutants (mainly #1 and #3) may have become deficient by a few per cent only. Some initiation defect was evident in Δ*seqA* cells since the distribution shifted towards cells with fewer chromosomes (except may be for the mutant #4; Figure B in S1 File). The initiation defects, however, do not seem to have affected the growth rates significantly (Table 1).

**Table 1:** Cell cycle parameters of *oriC*-*FRT* mutants with TaqI sites^a^.

TaqI site next to GATC #	Strain No.	Gen. Time^b^ (min)	Origin/Cell^c^	Cell mass^d^	Origin/Cell mass^e^	Asyn. Index^c^(%)	Frac. of uninitiatedCells^c^	Initiation mass
None (WT)	2239	33.4	3.49	1	1	3.9	0.20	0.39
#1	2240	34.6	3.43	0.99	0.99	12.4	0.19	0.39
#2	2241	33.6	3.48	0.96	1.04	7.2	0.20	0.37
#3	2242	31.6	3.33	1.05	0.91	14.5	0.19	0.40
#4	2243	33.7	3.49	0.97	1.03	4.1	0.19	0.37
#5	2244	32.9	3.28	0.97	0.97	5.9	0.24	0.38
#6	2245	34.1	3.34	1.01	0.95	9.6	0.22	0.39
#7	2246	32.7	3.48	1.01	0.99	8.8	0.18	0.39
#8	2247	33.6	3.31	0.99	0.95	2.4	0.28	0.40
#9	2248	32.7	3.40	1.06	0.92	5.0	0.22	0.42

^a^ Other than the generation time, cell cycle parameters were derived from Figure B in S1 File.

^b^Mean generation time from four biological replicates for WT was 33.5 ± 2.2 min. The generation times of the mutants did not vary significantly from that of the WT in pairwise comparisons (*p-*values 0.18–0.89).

^c^ Origin/cell, asyn. index and the fraction of uninitiated cells were determined from flow cytometry profiles after replication run-out. Origin/cell for WT among four biological replicates was 3.40 ± 0.09. The spread covers the range of values seen for the mutants. Further details are in Materials and Methods.

^d^ Cell mass is the average light scatter from flow cytometric analysis normalized to 1 for WT cells. The values varied about 3% among repeated measurements of the same sample and about 8% among biological replicates.

^e^ Values are normalized to 1 for WT cells.

### The hemimethylated DNA varies at different GATC sites of *oriC*

The two GATC sites in *oriC* that have overlapping restriction sites suitable for probing HM DNA (MboII and HphI sites, Fig 1B) showed enriched HM DNA when compared to several other GATC sites outside of *oriC* [28]. In the present study, the enrichment was not significantly affected upon addition of the *zeo* or the *FRT* marker (Figure A(C) in S1 File). When our *oriC* mutants were assayed using the TaqI enzyme, the HM DNA amount was enriched at all nine GATC sites within *oriC* compared to a distal site in *lacZ* (Fig 2A and 2B). The level of enrichment varied among the sites: it was maximal in the case of mutant #5 and minimal in the case of mutant #9, where the HM DNA level approached to that of the site in *lacZ*. The results were essentially identical when the *oriC* mutants had the *zeo* marker instead of the *FRT* site (Fig 2 and Figure C(A) in S1 File). In Δ*seqA* cells, the amount of HM DNA was less compared to the isogenic *seqA*^*+*^ cells and the extent of reduction was variable (Fig 2A and Figure C(A) in S1 File). These results indicate that SeqA sequesters with variable efficiency all the GATC sites of *oriC* with the possible exception of site #9.

Although restriction enzymes are sequence specific, the efficiency with which they digest their target sites can be context dependent [32, 33]. To address the possibility that intrinsic variation in cutting efficiency, rather than SeqA binding, could account for the variable HM DNA levels, we tested whether TaqI digestion efficiency varies among the sites in genomic DNA from *dam* minus cells. GATC sites are not methylated in *dam* minus cells and are not expected to interfere with TaqI digestion. The digestion efficiency of TaqI did vary somewhat among the TaqI sites of *oriC* but the average pattern was not correlated with the pattern seen in our experimentalt (*dam*+) strains (Fig 2 vs. Figure A(D) in S1 File). For example, the #5 site, which showed maximal sensitivity in *dam*+ cells, was not any more sensitive than others in *dam* minus cells. It appears that the intrinsic cutting efficiency of the sites is unlikely to account for the variability seen in Fig 2A.

We next considered whether competition from other proteins that interact with *oriC* could explain the variability in HM DNA level. Among the proteins that interact with *oriC*, only DnaA has multiple binding sites scattered throughout *oriC* [9]. In fact, the GATC sites probed in mutants #1, 2 and 5–9 have overlapping DnaA binding sites. These sites are low-affinity DnaA binding sites, most requiring DnaA to be complexed with ATP [9]. The two sites showing extreme HM DNA values (#5 and #9; Fig 2A) are both low affinity DnaA binding sites, suggesting that the variability in the affinity of DnaA for binding to its sites in *oriC* is unlikely to be the sole cause of the variability in HM DNA level that we observed across *oriC*.

Finally, efficient SeqA binding *in vitro* was shown to require two GATC sites phased by two to three integral helical turns of 10.4 bp [29, 30, 34]. The GATC sites of *oriC* are not separated by integral helical turns (Figure A(E) in S1 File). Specifically, the #5 GATC site where the HM DNA level was maximal is separated from its two flanking GATC sites by 18 and 17 bp. Similarly, the #9 site where the HM DNA level was minimal is also not explained by phasing requirements because it has on its right flank a GATC site nearly optimally separated by three helical turns (33 bp). SeqA cooperatively binds to DNA *in vitro*, and can also aggregate on DNA [30]. It remains to be seen whether these higher-order interactions and/or binding of other proteins that interact with *oriC* caused the local variation in HM DNA level.

### DnaA contributes to the HM DNA level at some of the GATC sites of *oriC*

**Fig 3:**
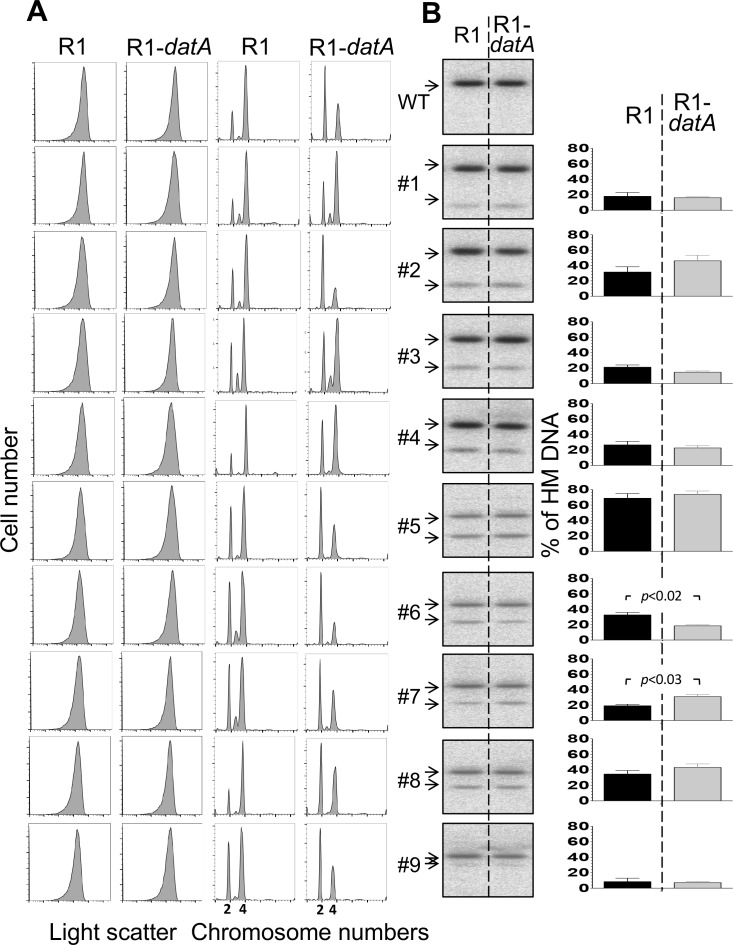
Effect of DnaA titration on cell size, initiation synchrony and HM DNA level at *oriC*. (A) Cell size and chromosome content of *FRT* marked *oriC* cells without (WT) or with mutations creating TaqI sites #1–9. The cells had an R1 or R1-*datA* plasmid that was used as a vector control or for titrating DanA-ATP, respectively. Cells were grown at 37°C in supplemented 1X M63 medium up to an OD_600_ ~ 0.15, and then processed either before or after replication run-out for flow cytometry to measure cell size by light scattering or chromosome content by fluorescence emission, respectively. The multimodal distribution of fluorescence profiles represent cells with different integral number of chromosomes as stated in the abscissa. (B) Southern blots of genomic DNA from cultures in (A) before replication run-out. Black and gray bars represent HM DNA levels in *seqA*^+^ strain in the presence of R1 and R1-*datA* plasmid, respectively. The details are otherwise same as in Fig 2A. Note that band intensities were not quantified for the WT as no cut band was seen or expected because WT *oriC* does not have a TaqI site in the probed region. The difference in HM DNA levels in the presence of R1 and R1-*datA* plasmids was considered statistically significant in the case of mutants #6 and #7 only (*P* < 0.05).

SeqA can interfere with binding of DnaA to some of its low-affinity sites [8]. In contrast, DnaA appears to aid sequestration since titration of DnaA-ATP by an R1-*datA* plasmid reduces the amount of HM *oriC* DNA at the MboII site [35]. We asked whether the same R1-*datA* plasmid could affect the HM DNA level at other GATC sites of *oriC*. Introduction of the plasmid to our *oriC*-*FRT*#1–9 strains reduced origin/cell-mass in WT and in five out of nine mutants (#2, #5–7 and #9; Fig 3A; Tables D vs. E in S1 File). The origin/cell-mass for the WT reduced from 1 to 0.82 and for the five mutants from 1.05 ± 0.11 to 0.80 ± 0.04 in the presence of the R1-*datA* plasmid compared to the isogenic cells carrying the R1 vector. This indicates that the R1-*datA* plasmid was equally effective in reducing initiation efficiency of these mutants as it is for the WT origin. The reduction was expected from earlier studies, since DnaA-ATP level is crucial for *oriC* activity [36]. Why reduction was seen in only some of the mutants remains to be understood. The asynchrony indices reduced mainly in the mutants where the origin/cell-mass reduced. In other words, initiation synchrony was not disturbed any more than what cannot be explained by initiation defficiency. (Tables D vs. E in S1 File). In the Δ*seqA* background, all the mutants showed asynchrony and overinitiation, but the effect of DnaA titration on the mutants was not estimated because of large asynchrony (Figure D(A) in S1 File).

To test the effect of the R1-*datA* plasmid on the HM DNA level, we assayed the amount of HM DNA from steady state cultures (Fig 3B). The effect of the plasmid was minimal in most cases, with the exception of mutant #6 in which HM DNA decreased, and mutant #7 in which the HM DNA increased. These results indicate that DnaA can help or hinder SeqA binding, depending upon the location of its binding site in *oriC*. While the increase in HM DNA in mutant #7 could result from reduced competition from DnaA, the decrease in mutant #6 may indicate that SeqA binding requires cooperation from DnaA. Alternate possibilities have also been considered in the Discussion. In the Δ*seqA* background, there was less origins/cell in the presence of the R1-*datA* plasmid compared to the presence of the R1vector for essentially all the mutants, indicating that titrating DnaA becomes more effective under over-replicating conditions (Figure D in S1 File).

### Contribution of individual GATC sites to initiation synchrony and *oriC* sequestration

**Fig 4:**
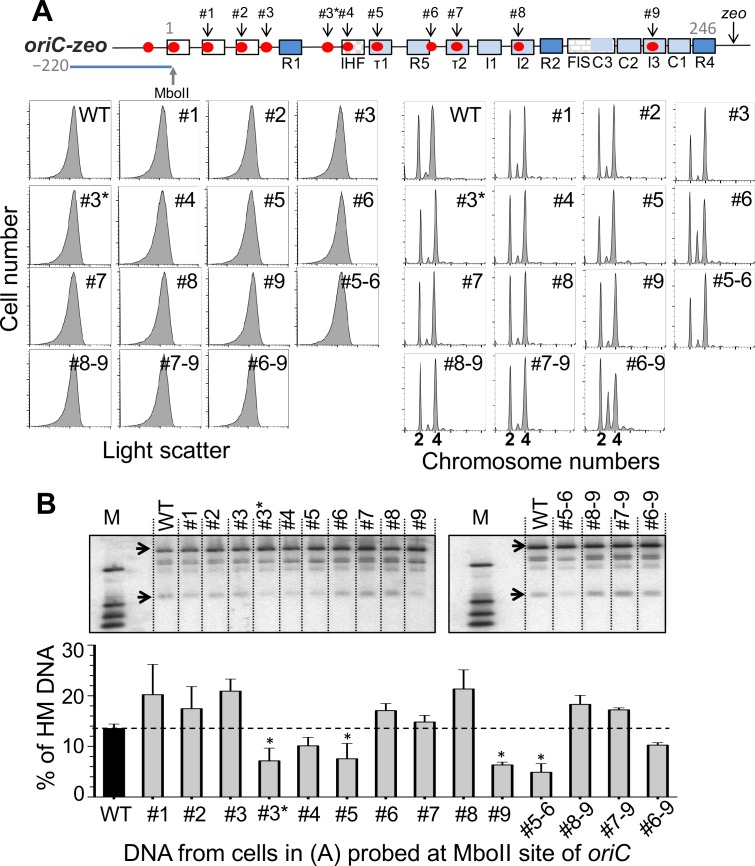
Effect of GATC mutations in *oriC* on initiation synchrony and the level of HM DNA at the MboII site of *oriC*. (A) The top line shows a schematic map of *oriC* as in Fig 1B except for an additional GATC site (#3*) included in this study. In these experiments *oriC* was marked with a *zeo* drug^R^ cassette. GATC sites at positions #1–9 and #3* were individually mutated to GTTC and the mutant cells were analyzed by flow cytometry as in Fig 3A before and after replication run-out. Cells were also studied after combining some of the mutations (#5–6 etc.). (B) HM DNA levels as in Fig 2A except that the levels were measured at the MboII site for all. The black bar represents the HM DNA level in the WT strain (*zeo* marked but without any GATC mutation within *oriC*) and the grey bars the same strain with individual or multiple GATC mutations. The error bars were determined as in Fig 2B. Mutants whose HM DNA levels were significantly different from the WT (*p-*value <0.05) are marked by asterisks. The dashed line provides a visual aid for comparing HM DNA levels in different mutants relative to the WT.

The results so far indicate that despite the variation in HM DNA across *oriC*, sequestration is nevertheless efficient enough to confer initiation synchrony (Fig 2 and Figure B in S1 File). This suggests that control by sequestration is rather robust and may not require efficient SeqA binding across all its sites in *oriC*. To test this inference further, we mutated individual GATC sites of *oriC* to GTTC and measured the effect on initiation synchrony and *oriC* sequestration (Fig 4A). Out of the ten mutated GATC sites that we tested, only the change within the R5 site (mutant #6) disturbed initiation synchrony. This particular change did not affect sequestration of *oriC* as measured at the natural MboII site (Fig 4B). GATC mutations at three other positions (#3*, #5 and #9), however, did reduce *oriC* sequestration without affecting initiation synchrony and growth rate (Table 2). Except for the #6 GATC mutant, these data support the view that sequestration is a robust process that does not require any one of the remaining GATC sites of *oriC* to be intact to support the extent of sequestration necessary for initiation synchrony.

**Table 2:** Cell cycle parameters of *oriC*-*zeo* mutants with mutated GATC^a^

*oriC-zeo* with mutated GATC	StrainNo.	Gen. Time(min)	Origin/Cell	Cell Mass	Origin/Cell Mass	Asyn. Index (%)	Frac. of Uninitiat.cells	Initiat. Mass
None (WT)	2073	33.2	3.28	1.0	1.0	4.3	27.5	0.40
#1GATC→GTTC	2901	32.1	3.13	1.07	0.89	11.6	29.7	0.41
#2 ,, → ,,	2902	32.8	3.16	1.08	0.89	10.2	29.8	0.41
#3 ,, → ,,	2903	32.0	3.22	1.02	0.96	5.3	23.4	0.39
#3* ,, → ,,	2916	31.7	2.99	1.06	0.85	5.7	25.2	0.40
#4 ,, → ,,	2904	32.7	3.04	1.06	0.87	6.3	29.9	0.41
#5 ,, → ,,	2905	32.2	3.42	1.03	1.01	5.8	22.2	0.39
#6 ,, → ,,	2906	32.3	2.94	1.05	0.85	20.2	27.1	0.40
#7 ,, → ,,	2907	32.1	3.26	1.03	0.97	7.3	28.4	0.40
#8 ,, → ,,	2908	32.0	2.98	1.03	0.88	5.3	30.7	0.41
#9 ,, → ,,	2909	34.3	3.06	1.01	0.92	5.1	29.0	0.41
#5–6 ,, → ,,	2910	33.2	2.87	1.03	0.85	3.3	23.2	0.39
#8–9 ,, → ,,	2911	35.1	2.89	1.07	0.82	5.0	29.7	0.41
#7–9 ,, → ,,	2912	35.9	2.95	1.04	0.87	6.0	29.2	0.41
#6–9 ,, → ,,	2913	35.1	2.71	1.04	0.80	19.6	26.3	0.40
#6 ,, →GATG	2914	34.6	3.21	1.06	0.92	5.0	31.4	0.41
#6 ,, →GAAC	2915	34.7	3.44	1.08	0.97	6.7	19.8	0.38

^a^ Other than the generation times, cell cycle parameters were derived from Figs 4 and 5.

**Fig 5:**
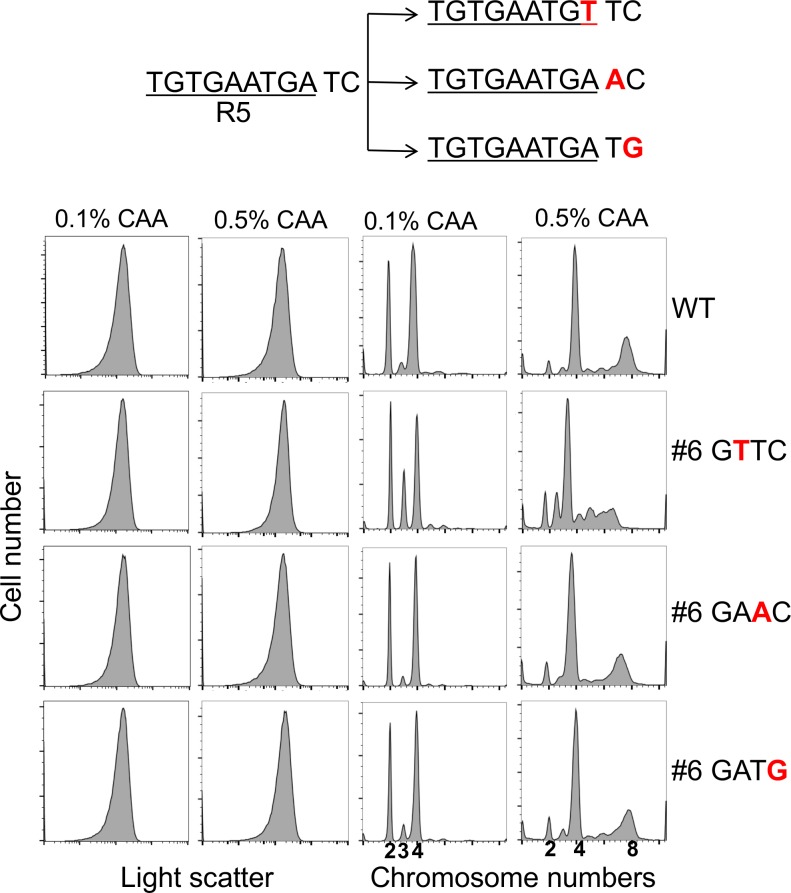
Importance of integrity of the DnaA binding site R5 and not its methylation in initiation synchrony. The overlapping GATC site of R5 was mutated at three positions (shown in red), two of which reside outside of the canonical 9-mer DnaA binding site (underlined). Cell sizes and chromosome contents of the above GATC mutants were determined as in Fig 4A, using two different concentrations of casamino acids (CAA).

Since the adenine residue of the #6 GATC site is part of the R5 DnaA binding site, we noted that the asynchrony phenotype (20%; Table 2) of this particular mutant could result from a defect in DnaA binding rather than the lack of SeqA binding. To address this possibility, we mutated #6 GATC to GAAC or to GATG (Fig 5). Both of these base changes are outside of the canonical 9-bp DnaA binding site and therefore should abrogate methylation of the site without affecting the R5 DnaA binding site. These new GATC mutants no longer showed the asynchrony phenotype, suggesting that a defect in DnaA binding, rather than in SeqA binding, caused asynchrony when GATC was changed to GTTC in R5.

### SeqA binding to the last three GATC sites (#7-#9) of *oriC* has the potential to control initiation

The importance of SeqA binding to the left half of *oriC* was previously examined by simultaneously mutating either three or seven GATC sites (up to the site in R5) [37]. Both sets of changes caused replication asynchrony and reduced HM DNA to nearly the level observed in Δ*seqA* cells, particularly evident in the mutant with seven changes. This suggested that the last three GATC sites (#7–9) in the right half of *oriC* do not significantly contribute to the sequestration required for initiation synchrony. When we combined the GTTC mutations in the last three GATC sites (#7–9), the triple mutant neither compromised synchrony nor reduced HM DNA at the MboII site (Fig 4A and 4B). However, when the triple mutant was combined with the #6 GATC mutation, the quadruple mutant with changes in #6–9 GATC sites showed reduced HM DNA and improved initiation efficiency at faster growth rates in the presence of 0.5% CAA, although the initiation remained asynchronous (Fig 4B and Figure E in S1 File). It appears that the last three GATC sites have the potential to negatively control initiation, but this becomes apparent only when the initiation is partially compromised. The initiation defect of the #6 mutant was also alleviated when mutations in the #5 and #6 GATC sites were combined (Fig 4 and Figure E in S1 File). Thus, although mutating individual GATC sites does not result in an initiation defect, eliminating SeqA binding even to a single site can help when the initiation efficiency is already suboptimal.

## Discussion

During rapid growth when *E*. *coli* maintains multiple origins, sequestration of newly replicated origins is required for synchronous initiation from all the origins in a narrow window of time. Sequestration is mediated by binding of SeqA protein to GATC sites of the origin (*oriC*) when they are hemi-methylated (HM), as in newly replicated origins. To understand the process of sequestration better, we have individually probed most of the GATC sites of *oriC* for SeqA binding. SeqA is found to bind to all the GATC sites tested, indicating that sequestration normally involves binding to the full complement of GATC sites in *oriC*. However, synchronous initiation is still retained when the GATC sites were mutated individually, indicating the robustness of the sequestration process.

SeqA binding *in vivo* is inferred if it can prolong the HM state of a GATC site. By this criterion, SeqA binds to different GATC sites of *oriC* to different extents. Efficient SeqA binding *in vitro* requires cooperation from adjacent GATC sites that are on the same face of the DNA and phased by about three helical turns of B-DNA [21, 29, 30, 34]. One explanation for variable SeqA binding to different GATC sites is that the GATC sites in *oriC* are not phased by integral helical turns. The variation can also be rationalized by assuming that SeqA competes with many other proteins that bind to *oriC*. We tested the effect of titrating DnaA, the major protein that binds to *oriC*, on the HM DNA level. Significant but contrasting changes were seen at only two of the GATC sites. Both the observed increase at one site (mutant #6) and the decrease at the other (mutant #7) indicate that DnaA can cooperate as well as compete with SeqA binding, as was suggested earlier [8, 35]. The altered sequestration levels, however, did not change initiation efficiency (mutants #6 and #7, Fig 3A and 3B). Initiation thus seems buffered against small changes in sequestration.

The cooperation from DnaA for SeqA binding to #6 GATC site may not be the only explanation of our data. In the present study, DnaA was titrated using a R1-*datA* plasmid. The *datA* site reduces the availability of DnaA-ATP, thereby delay replication initiation [38]. The initiation delay will reduce the number of cells in the culture undergoing replication and, thereby, in the sequestration period. In this scenario the HM DNA level would also lower without a direct role of DnaA in controlling SeqA binding. The question, however, remains is why this scenario did not reduce HM DNA level at other sites. The presence of R1-*datA* plasmid also derepresses the *dnaA* promoter and increases the overall DnaA level [36]. The *dnaA* promoter is also sequestered like *oriC* because of the presence of six GATC sites in the region. However, when the GATC sites in the promoter region are mutated the initiation synchrony phenotype still persists, indicating that fluctuations in DnaA concentration are not critical for the phenotype [39].

The robustness of sequestration was most evident when we individually inactivated the GATC sites of *oriC*. The mutants with single mutated GATC sites showed normal level of initiation and initiation synchrony. Together with varying level of SeqA binding to different GATC sites (Fig 2A), these results suggest against cooperativity in SeqA binding. (We cannot rule out the possibility that because of cooperativity the mutated sites were still bound by SeqA.) There is also indication against cooperative binding since SeqA binding to one site does not seem to influence binding to neighboring sites [8]. The cooperativity, however, has been indicated *in vitro* [30]. Inactivation of the DnaA binding site I2 by mutation of GATC to GATT no longer allows SeqA to interfere with DnaA binding to I2, but SeqA still prevents DnaA binding to a distal site R5. Similarly, when a GATC was engineered into the R1 site, SeqA blocked DnaA binding to that site only.

Initiation synchrony was disturbed when the #6 GATC was changed to GTTC in the R5 DnaA binding site (mutant#6, Fig 4A). We believe that reduced DnaA binding, rather than lack of SeqA binding, is the basis of the observed asynchrony. SeqA binding is known to prevent DnaA binding to some of the lower affinity DnaA binding sites [40]. This is clearly the case both *in vivo* and *in vitro* in four of the low-affinity DnaA binding sites tested, including R5 (τ2, R5, I2 and I3) [8]. Reduced DnaA binding upon elimination of SeqA binding to R5 was therefore unexpected. This led us to speculate that the GTTC change reduced DnaA binding and thereby compromised initiation synchrony. Indeed, initiation synchrony was regained when the GATC was mutated to selectively affect SeqA binding (Fig 5). It is intriguing that among the many mutations that we have created in *oriC*, only the one in R5 showed a significant synchrony defect. R5 could be playing a more important role in initiation than other low-affinity DnaA binding sites, as we discuss below.

Chromosomal replication is disturbed mildly when individual protein binding sites of *oriC* are mutated along with inversion and scrambling of the R5 site sequence [41, 42]. However, compared to scrambling of R2 or R3, scrambling of the R5 sequence reduced replication more significantly and increased the asynchrony index about two-fold (from 10 to 20) [41]. R5 was also scrambled without disturbing the GATC site [43]. However, this was not sufficient for *oriC* activity in a plasmid-based replication assay, suggesting that binding of DnaA to R5 is required for initiation. It is also consistent that a viable minimal origin requires the R5 site, although the last 83 bp of *oriC* that includes the sites from R2 to R4 could be deleted [44]. More direct evidence for the importance of R5 was observed in an *in vitro* reconstituted system for origin opening [15]. In this assay, the opening could be obtained even when all *oriC* sequences beyond R5 were deleted, but not when the deletion was extended to include R5. This suggests that the nucleoprotein complex that opens DNA requires DnaA binding to R5. R5 was also required to nucleate DnaA-ATP binding to three weaker downstream sites τ2, I1 and I2 [9]. This cooperative binding requires the sites to be oriented in the same direction, explaining why previous attempts to invert R5 resulted in a non-functional *oriC* in a plasmid based assay [43].

How could R5 be important? Cooperative interactions that allow DnaA binding to low-affinity sites to the right of R5 appears important, but this cannot be its only role because those sites can be deleted for origin opening, whereas R5 is still required [8, 15]. At present, the role in nucleo-protein complex formation at *oriC* conducive to origin opening may distinguish R5 from other low-affinity DnaA binding sites of *oriC*.

We also find that the GATC sites to the right of R5 (sites #7–9) play an insignificant role in synchrony even when they are made defective simultaneously. The three sites alone also do not prevent overinitiation when the upstream GATC sites are mutated [37]. The sites are phylogenetically conserved indicating that their presence is important [45]. The functional significance of the sites #7–9 became evident when the GATC mutation in R5 was combined with GATC mutations of sites #7–9. The latter mutations more than compensated for the initiation defect of the R5 mutation (Figure E in S1 File). In other words, the sequestration of the last three GATC sites can contribute negatively to the regulation of *oriC* although this contribution appears to be redundant when R5 is not mutated. Similarly, although mutating the GATC site #5 (in τ1) did not significantly change the initiation phenotype on its own, it did rescue the initiation defect of the mutation in R5. In other words, reducing sequestration of the #5 site was helpful only when the initiation efficiency of *oriC* was compromised. These results lead us to believe that there is excess SeqA binding capacity at *oriC* to ensure its important role in controlling initiation.

## Supporting Information

S1 File **Table A. Bacterial strains. Table B. Plasmids. Table C. Primers. Table D. Cell cycle parameters of *oriC*-*FRT* mutants with TaqI sites carrying R1 plasmid. Table E. Cell cycle parameters of *oriC*-*FRT* mutants with TaqI sites carrying R1-*datA*. Figure A. Construction of strains with TaqI sites overlapping the GATC sites within *oriC*.** (A) Restriction sites used for HM DNA analysis. Recognition sequences (in capital letters) of enzymes MboII and HphI naturally occur within *oriC*, and of TaqI were created in this study to overlap with the GATC sites. When fully methylated the sites resist enzyme digestion. Upon replication, one of the two sister sites become sensitive to digestion, the one in which the methylated adenine falls outside of the enzyme recognition sequence. (B) Schematic map of *oriC* as in Fig 1B marked with either an FRT site or a zeo cassette, inserted 27 bp away from the end of the R4 site. The primers (jj40+jj42) used to amplify the *oriC* region are shown by horizontal arrows at the two flanks of the origin, and the fragment used for probing Southern blots (blue line with dashed extension) at the left end of the origin is same as in Fig 2A. To verify the presence of TaqI sites, genomic DNA either FRT or Zeo marked *seqA*+ and Δ*seqA* cells was used to amplify the origin region by PCR, the amplified products were digested with TaqI and resolved on an 1.3% agarose gel. M represent mol. wt. markers (NEB) and WT represent cells without any TaqI site within minimal *oriC* sequence. The presence of a TaqI site is indicated if the upper band is split into two smaller bands. The change in the relative sizes of the digested bands confirm shifting positions of the TaqI site created within *oriC* in mutants #1–9. Note that PCR products are not methylated, thus are fully sensitive to TaqI digestion. (C) Comparison of HM DNA level in *oriC*, *oriC*-FRT and *oriC*-zeo strains that are either *seqA*+ or Δ*seqA*. The genomic DNA of the strains was digested with either MboII or HphI. Other details for probing and quatification of HM DNA were as described in Fig 2A. (D) Relative TaqI sensitivity of FRT marked genomic DNA from dam minus derivatives of *oriC* mutants #1–9. The genomic DNA was partially digested at 55°C for 10 min to monitor relative sensitivity of the TaqI sites created within *oriC* to TaqI digestion, otherwise the details are same as in Fig 2A. The band of interest (the lowest band of the gel) is generated by digestion of one TaqI site within *oriC* and the other in the left flank of *oriC*, and its intensity was quantified with respect to all other bands of the gel (the panel on the right). The mean intensity from three independent DNA preparations is shown with one standard deviation of the mean. (E) Distance (bp) separating different GATC sites of *oriC*. The distances are shown below the schematic map of *oriC* as in Fig 1B. **Figure B. DNA histogram of FRT marked isogenic *seqA***^**+**^ **and Δ*seqA* strains of *oriC* mutants #1–9.** These experiments were done as in Fig 3A except these cells did not have the R1 plasmids. **Figure C. HM DNA level at different GATC sites of *oriC* in *seqA***^**+**^ **and Δ*seqA* strains.** The experiments in (A) and (B) were done similarly to those in Fig 2A and 2B, except the strains were marked with *zeo* in place of the *FRT* site. **Figure D. Effect of DnaA titration on initiation synchrony and HM DNA level at *oriC*.** The experiments in (A) and (B) were done similarly to those in Fig 3A and 3B, except that the strains were Δ*seqA* derivatives of those used in Fig 3. **Figure E. Overcoming of initiation defect due to a mutation in #6 GATC in R5 by GATC mutations in #5 or #7–9 GATC of *oriC*.** Chromosome contents of the GATC mutants were determined as in Fig 4A, except that CAA concentration was 0.5% instead of 0.1%. Note that initiation becomes more efficient in the double mutant #5–6 and in the quadruple mutant #6–9 compared to the single mutant #6.
(PDF)
