# Supplementary material for: Inactivation of Individual SeqA Binding Sites of the E. coli Origin Reveals Robustness of Replication Initiation Synchrony
Source: PLoS One. 2016 Dec 8;11(12):e0166722. doi: 10.1371/journal.pone.0166722 (PMC5145175; doi:10.1371/journal.pone.0166722)
Supplement: S1 File — Table A. Bacterial strains. Table B. Plasmids. Table C. Primers. Table D. Cell cycle parameters of oriC-FRT mutants with TaqI sites carrying R1 plasmid. Table E. Cell cycle parameters of oriC-FRT mutants with TaqI sites carrying R1-datA. Figure A. Construction of strains with TaqI sites overlapping the GATC sites within oriC. (A) Restriction sites used for HM DNA analysis. Recognition sequences (in capital letters) of enzymes MboII and HphI naturally occur within oriC, and of TaqI were created in this study to overlap with the GATC sites. When fully methylated the sites resist enzyme digestion. Upon replication, one of the two sister sites become sensitive to digestion, the one in which the methylated adenine falls outside of the enzyme recognition sequence. (B) Schematic map of oriC as in Fig 1B marked with either an FRT site or a zeo cassette, inserted 27 bp away from the end of the R4 site. The primers (jj40+jj42) used to amplify the oriC region are shown by horizontal arrows at the two flanks of the origin, and the fragment used for probing Southern blots (blue line with dashed extension) at the left end of the origin is same as in Fig 2A. To verify the presence of TaqI sites, genomic DNA either FRT or Zeo marked seqA+ and ΔseqA cells was used to amplify the origin region by PCR, the amplified products were digested with TaqI and resolved on an 1.3% agarose gel. M represent mol. wt. markers (NEB) and WT represent cells without any TaqI site within minimal oriC sequence. The presence of a TaqI site is indicated if the upper band is split into two smaller bands. The change in the relative sizes of the digested bands confirm shifting positions of the TaqI site created within oriC in mutants #1–9. Note that PCR products are not methylated, thus are fully sensitive to TaqI digestion. (C) Comparison of HM DNA level in oriC, oriC-FRT and oriC-zeo strains that are either seqA+ or ΔseqA. The genomic DNA of the strains was digested with either MboII or HphI. Other details [file pone.0166722.s001.pdf]

Inactivation of Individual SeqA Binding Sites of the *E. coli* Origin Reveals Robustness of Replication Initiation Synchrony

Jyoti K Jha, Dhruba K Chattoraj

Supporting Information S File

Table A. Bacterial strains.

Name	Genotype	Drug^R	Number	Reference
MG1655	*E. coli* K-12 F^- λ^- *ilvG rfb-50 rph-1*		BR1703	[1]
MG1655 *ΔseqA10*	BR1703 *ΔseqA10*		BR1704	[2]
MG1655 mini-λ Tet	BR1703 mini-λ Tet	Tc	CVC1394	[3]; Figs 4 and 5; Figures A-C
MG1655 *ΔseqA10* mini-λ Tet	BR1704 mini-λ Tet	Tc	CVC2064	Figures A-C
Δ*dam*::*Tn9*	NS2655 *gal*Δ*dam*::Tn9 λi21 P1:7 *cI*+ *galET*Δ*K*	Cm	BR2786	N. Sternberg; Figure A
oriC-zeo	BR1703 *oriC-zeo*	Zeo	CVC2073	Figs 4 and 5; Figures A and C
,, #1	,, #1		CVC2142	Figures A and C
,, #2	,, #2		CVC2143	
,, #3	,, #3		CVC2144	
,, #4	,, #4		CVC2145	
,, #5	,, #5		CVC2150	
,, #6	,, #6		CVC2151	
,, #7	,, #7		CVC2152	
,, #8	,, #8		CVC2153	
,, #9	,, #9		CVC2075	
*oriC-zeo*Δ*seqA*	BR1704 *oriC-zeo*	Zeo	CVC2092	Figures A and C
,, #1	,, #1		CVC2146	
,, #2	,, #2		CVC2147	
,, #3	,, #3		CVC2148	
,, #4	,, #4		CVC2149	
,, #5	,, #5		CVC2154	
,, #6	,, #6		CVC2155	
,, #7	,, #7		CVC2156	
,, #8	,, #8		CVC2157	
,, #9	,, #9		CVC2094	
oriC-FRT	BR1703 *oriC-FRT*		CVC2239	Figs 2 and 3; Figures A and B
,, #1	,, #1		CVC2240	
,, #2	,, #2		CVC2241	
,, #3	,, #3		CVC2242	
,, #4	,, #4		CVC2243	

,, #5	,, #5		CVC2244	
,, #6	,, #6		CVC2245	
,, #7	,, #7		CVC2246	
,, #8	,, #8		CVC2247	
,, #9	,, #9		CVC2248	
oriC-FRTΔseqA	BR1704 *oriC-FRT*		CVC2249	
,, #1	,, #1		CVC2250	
,, #2	,, #2		CVC2251	
,, #3	,, #3		CVC2252	
,, #4	,, #4		CVC2253	Fig 2: Figures A, B and D
,, #5	,, #5		CVC2254	
,, #6	,, #6		CVC2255	
,, #7	,, #7		CVC2256	
,, #8	,, #8		CVC2257	
,, #9	,, #9		CVC2258	
oriC-zeo GATC→GTTC #1	CVC2073 GATC→GTTC #1		CVC2901	
,, #2	,, #2		CVC2902	
,, #3	,, #3		CVC2903	Fig 4: Figure E
,, #4	,, #4		CVC2904	
,, #5	,, #5		CVC2905	
,, #6	,, #6		CVC2906	Figs 4 and 5; Figure E
,, #7	,, #7	Zeo	CVC2907	
,, #8	,, #8		CVC2908	
,, #9	,, #9		CVC2909	
,, #5-6	,, #5-6		CVC2910	Fig 4: Figure E
,, #8-9	,, #8-9		CVC2911	
,, #7-9	,, #7-9		CVC2912	
,, #6-9	,, #6-9		CVC2913	
oriC-zeo GATC→GATG #6	CVC2073 GATC→GATG #6		CVC2914	Fig 5
oriC-zeo GATC→GAAC #6	CVC2073 GATC→GAAC #6		CVC2915	

Table B. Plasmids.

Name	Genotype	Drug^R	Reference
pEM7-Zeo			Invitrogen
pJJ04	pEM7-*zeo*-*oriC*(3925738-4038)		
pJJ06	pJJ04 GA→TC(3925759-60) #1		
pJJ40	,, AA→TC(3925774-75) #2		
pJJ41	,, AG→TC(3925787-88) #3		
pJJ46	,, GT→TC(3925827-28) #4	Ap, Zeo	Figs 2 and 3; Figures A, B, and D
pJJ47	,, AG→TC(3925845-46) #5		
pJJ48	,, AT→TC(3925863-64) #6		
pJJ49	,, GT→TC(3925870-71) #7		
pJJ55	,, GG→TC(3925892-93) #8		
pJJ07	,, TT→TC(3925960-61) #9		
pJJ385	pJJ04 GATC→GTTC #1		
pJJ386	,, GATC→GTTC #2	Ap, Zeo	Fig 4

pJJ387	„ GATC→GTTC #3		
pJJ388	„ GATC→GTTC #3*		
pJJ389	„ GATC→GTTC #4		
pJJ379	„ GATC→GTTC #5		
pJJ391	„ GATC→GTTC #6		
pJJ369	„ GATC→GTTC #7		
pJJ370	„ GATC→GTTC #8		
pJJ371	„ GATC→GTTC #9		
pJJ372	„ GATC→GTTC #8-9		Figs 2 and 3; Figures A and B
pJJ374	„ GATC→GTTC #7-9		
pJJ377	„ GATC→GTTC #6-9		
pJJ390	„ GATC→GTTC #5-6		
pJJ415	„ GATC→GATG #6		
pJJ416	„ GATC→GAAC #6		
pJEL109	MiniR1	Ap	[4]; Fig 3; Figure D
pMOR1	MiniR1-*datA*	Ap	[5]; Fig 3; Figure D
pRFB105	pUC *ori*	Ap, Kn	Figs 2 and 3; Figure D
pSIM5	pSC101 *ori*; Carries λ red genes	Ap	[6]; Figs 2 and 3; Figure D
pCP20	Supplies flp recombinase	Ap, Cm	[7]; Figs 2 and 3; Figure D
mini-λ tet	Carries λ red genes	Tc	[3]

Table C. Primers

Name	Sequence	Used in
jj07	AGATCTATTTATTTATCGATCTGTTCTATTGTGATC	pJJ06
jj08	ACAATAGAACAGATCGATAAATAAATAGATCTTCT	
jj09	GGATAACTACCGGTCGATCCAAGCTTCCTGACA	pJJ07
jj10	GGAAGCTTGGATCGACCGGTAGTTATCCAAAGA	
jj15	CTTAGGATCCAGACCTGGGATCCTGGGTAT	pJJ04
jj16	TTAGGAATTCAATAAGTATACAGATCGTGCG	
jj21	TGGGATCGTGGGTTAATTTACTCAAATAAGTATACAGATCGCGATATCGCTAGCTCGAGCACG	transfer of *oriC* mutants
jj35	GGATTCTACTCAACTTTGTCGGCTTGAGAAAGACCTGGGATCCTGGGTATTAAAAAGA	
jj40	GATATTGTGTGTCAAAAGCAGAGTCT	*oriC* probe
jj41	GGATCCCAGGTCTTTCTCAAGCCGA	
jj42	GAGGCAGAACTCAAAAATTCCGGTG	*oriC* flanking primer
jj81	CTGTTCTATTTCGATCTCTTATTAGGATCG	pJJ40
jj82	CTAATAAGAGATCGAAATAGAACAGATCTC	
jj85	TTGTGATCTCTTATTTCGATCGCACTGCCCTGTGG	pJJ41
jj86	CACAGGGCAGTGCGATCGAAATAAGAGATCACAATAG	
jj88	CTTATCCACAAAGATCGAGCTCCTTAATAGTAGATCT	
jj93	CAAGGATCCGGCTTTTTCGATCAACAACCTGGAAAGG	pJJ46
jj94	TCCAGGTTGTTGATCGAAAAAGCCGGATCCTTGTTATC	
jj97	GATCAACAACCTGGAATCGATCATTAACTGTGAATG	pJJ47
jj98	TTCACAGTTAATGATCGATTCCAGGTTGTTGATCT	
jj101	GGATCATTAACTGTGATCGATCGGTGATCCTGGACCG	pJJ48
jj102	CCAGGATCACCGATCGATCACAGTTAATGATCC	

jj105	AACTGTGAATGATCGTCGATCCTGGACCGTATAAGC	pJJ49
jj106	CTTATACGGTCCAGGATCGACGATCATTCACAGTTAATG	
jj107	CTGGACCGTATAAGCTTCGATCAGAATGAGGGGTTATAC	pJJ54
jj108	ACCCCTCATTCTGATCGAAGCTTATACGGTCCAGG	
jj122	TCCGGATAAAACATGGTGATTGC	*oriC* sequencing
jj168	ATCATCAGGTTCGGTTGGTTCTC	*oriC* probe for HphI HM detection
jj169	ATGTTTTATCCGGATCCTTTTGAC	
jj184	ACAGAGTTATCCACAGTAGATCGCACGATCTGTATACTTATTTA TCCTCCTTAGTTCCTATTCC	For amplifying *FRT-Kn-FRT* cassette
jj185	ATCCGGCAGAAGAATGGCTGGGATCGTGGGTTAATTTACTCATT GTGTAGGCTGGAGCTGCTTC	
jj193	CCTGTATGTGGTGGATGAAGC	*lacZ* probe
jj194	CAGATTTGATCCAGCGATAC	
jj507	GAATGATCGGTGTTCCTGGACCGTATAAGCTG	pJJ369, 373, 374
jj508	ACGGTCCAGGAACACCGATCATTCACAGTTAATG	
jj509	CCGTATAAGCTGGGTTCAGAATGAGGGGTTATAC	pJJ370
jj510	CCTCATTCTGAACCCAGCTTATACGGTCCAG	
jj511	TAACTACCGGTTGTTCCAAGCTTCCTGACAGAG	pJJ371, 372
jj512	CAGGAAGCTTGGAACAACCGGTAGTTATCCAAAG	
jj522	CAACCTGGAAAGGTTCATTAACTGTGAATG	pJJ379
jj523	CAGTTAATGAACCTTTCCAGGTTGTTGATC	
jj529	TAACTGTGAATGTTCGGTGATCCTGGACCG	pJJ390,391
jj530	AGGATCACCGAACATTCACAGTTAATGA	
jj531	CTATTTATTTAGAGTTCTGTTCTATTGTGATCTC	pJJ385
jj532	CACAATAGAACAGAACTCTAAATAAATAGATCTTC	
jj533	GTTCTATTGTGTTCTCTTATTAGGATCGCAC	pJJ386
jj534	TCCTAATAAGAGAACACAATAGAACAGATCTC	
jj535	CTCTTATTAGGTTCGCACTGCCCTGTGGA	pJJ387
jj536	GGCAGTGCGAACCTAATAAGAGATCACAATAG	
jj537	GTGGATAACAAGGTTCCGGCTTTTAAGATCAAC	pJJ388
jj538	CTTAAAAGCCGGAACCTTGTTATCCACAGGGC	
jj554	GTGAATGATGGGTGATCCTGGACCGTATAAG	pJJ415
jj555	CAGGATCACCCATCATTCACAGTTAATG	
jj556	TAACTGTGAATGAACGGTGATCCTGGACCG	pJJ416
jj557	CCAGGATCACCGTTCATTCACAGTTAATG	

Table D. Cell cycle parameters of *oriC-FRT* mutants with TaqI sites carrying R1 plasmid[a].

TaqI site next to GATC	Gen. Time (min)	Origin/ Cell	Cell Mass	Origin/ Cell Mass	Asyn. Index (%)	Frac. of Uninitiated Cells	Initiation Mass
None (WT)	32.2	3.05	1	1	2.9	0.13	0.38
#1	32.6	3.09	0.97	1.05	9.3	0.13	0.36
#2	33.1	3.18	0.93	1.12	6.9	0.19	0.41
#3	31.9	3.0	0.93	1.05	14.9	0.21	0.41
#4	32.6	3.05	0.96	1.03	3.5	0.15	0.36
#5	31.8	2.99	1.05	0.93	3.5	0.24	0.40
#6	30.5	2.96	0.97	1.0	10.2	0.26	0.37
#7	32.5	2.92	0.94	1.01	10.3	0.27	0.44
#8	32.6	2.97	0.92	1.05	4.8	0.13	0.36
#9	34.4	3.33	0.90	1.21	3.6	0.18	0.36

[a] Other than the generation times, cell cycle parameters were derived from Fig 3A.

Table E. Cell cycle parameters of *oriC-FRT* mutants with TaqI sites carrying R1-*datA*[a].

TaqI site next to GATC	Gen. Time (min)	Origin/ Cell	Cell Mass	Origin/ Cell Mass[b]	Asyn. index (%)	Frac. of Uninitiated Cells	Initiation Mass
None (WT)	36.3	2.55	1.02	0.82	1.7	0.45	0.41
#1	35.5	3.14	0.93	1.11	9.1	0.20	0.37
#2	37.4	2.28	0.99	0.76	3.2	0.54	0.43
#3	34.1	2.90	0.97	0.98	11.6	0.24	0.38
#4	34.1	3.03	0.88	1.13	3.5	0.26	0.41
#5	34.4	2.48	0.99	0.82	2.1	0.44	0.39
#6	31.5	2.33	1.05	0.73	3.6	0.53	0.45
#7	32.7	2.57	1.02	0.83	4.2	0.40	0.41
#8	32.3	3.01	0.99	1.00	3.5	0.39	0.38
#9	37.7	2.77	1.07	0.85	3.4	0.40	0.46

[a] Other than the generation times, cell cycle parameters were derived from Fig 3A.

[b] Values are normalized to 1 for the WT with R1 carrying cells of Table D.

1. Blattner FR, Plunkett G, 3rd, Bloch CA, Perna NT, Burland V, Riley M, et al. The complete genome sequence of *Escherichia coli* K-12. Science. 1997;277(5331):1453-74.

2. Slater S, Wold S, Lu M, Boye E, Skarstad K, Kleckner N. *E. coli* SeqA protein binds *oriC* in two different methyl-modulated reactions appropriate to its roles in DNA replication initiation and origin sequestration. Cell. 1995;82(6):927-36.

3. Court DL, Swaminathan S, Yu D, Wilson H, Baker T, Bubunenko M, et al. Mini-lambda: a tractable system for chromosome and BAC engineering. Gene. 2003;315:63-9.

4. Lϕbner-Olesen A, Boye E, Marinus MG. Expression of the *Escherichia coli dam* gene. Mol Microbiol. 1992;6(13):1841-51.

5. Morigen, Boye E, Skarstad K, Lϕbner-Olesen A. Regulation of chromosomal replication by DnaA protein availability in Escherichia coli: effects of the *datA* region. Biochim Biophys Acta. 2001;1521(1-3):73-80.

6. Datta S, Costantino N, Court DL. A set of recombineering plasmids for gram-negative bacteria. Gene. 2006;379:109-15.

7. Datsenko KA, Wanner BL. One-step inactivation of chromosomal genes in *Escherichia coli* K-12 using PCR products. Proc Natl Acad Sci U S A. 2000;97(12):6640-5.

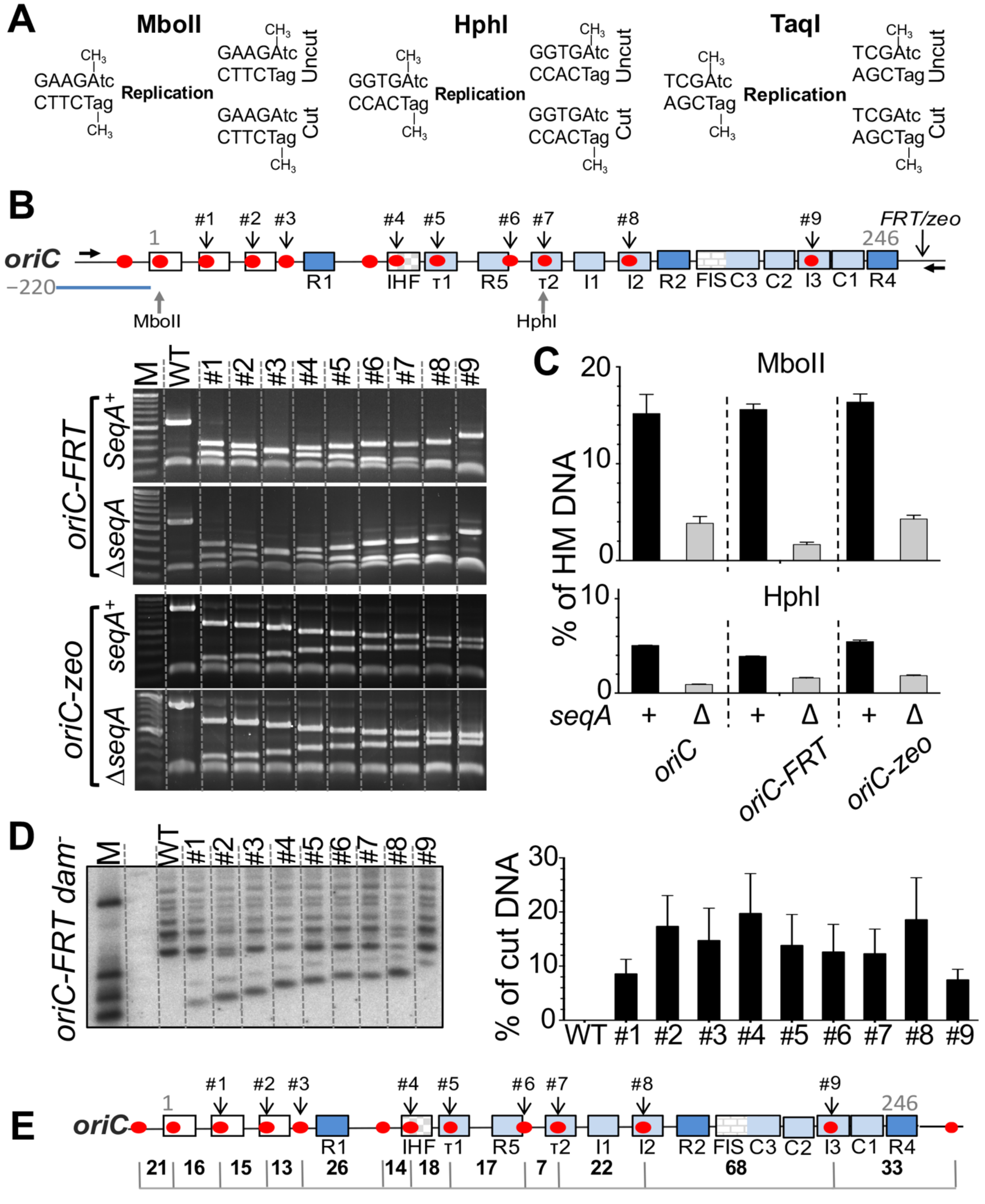

Figure A. Construction of strains with TaqI sites overlapping the GATC sites within *oriC*. (A) Restriction sites used for HM DNA analysis. Recognition sequences (in capital letters) of enzymes MboII and HphI naturally occur within *oriC*, and of TaqI were created in this study to overlap with the GATC

sites. When fully methylated the sites resist enzyme digestion. Upon replication, one of the two sister sites become sensitive to digestion, the one in which the methylated adenine falls outside of the enzyme recognition sequence. (B) Schematic map of *oriC* as in Fig 1B marked with either an FRT site or a zeo cassette, inserted 27 bp away from the end of the R4 site. The primers (jj40+jj42) used to amplify the *oriC* region are shown by horizontal arrows at the two flanks of the origin, and the fragment used for probing Southern blots (blue line with dashed extension) at the left end of the origin is same as in Fig 2A. To verify the presence of TaqI sites, genomic DNA either FRT or Zeo marked *seqA*+ and Δ*seqA* cells was used to amplify the origin region by PCR, the amplified products were digested with TaqI and resolved on an 1.3% agarose gel. M represent mol. wt. markers (NEB) and WT represent cells without any TaqI site within minimal *oriC* sequence. The presence of a TaqI site is indicated if the upper band is split into two smaller bands. The change in the relative sizes of the digested bands confirm shifting positions of the TaqI site created within *oriC* in mutants #1-9. Note that PCR products are not methylated, thus are fully sensitive to TaqI digestion. (C) Comparison of HM DNA level in *oriC*, *oriC*-FRT and *oriC*-zeo strains that are either *seqA*+ or Δ*seqA*. The genomic DNA of the strains was digested with either MboII or HphI. Other details for probing and quatification of HM DNA were as described in Fig 2A. (D) Relative TaqI sensitivity of FRT marked genomic DNA from dam minus derivatives of *oriC* mutants #1-9. The genomic DNA was partially digested at 55^0C for 10 min to monitor relative sensitivity of the TaqI sites created within *oriC* to TaqI digestion, otherwise the details are same as in Fig 2A. The band of interest (the lowest band of the gel) is generated by digestion of one TaqI site within *oriC* and the other in the left flank of *oriC*, and its intensity was quantified with respect to all other bands of the gel (the panel on the right). The mean intensity from three independent DNA preparations is shown with one standard deviation of the mean. (E) Distance (bp) separating different GATC sites of *oriC*. The distances are shown below the schematic map of *oriC* as in Fig 1B.

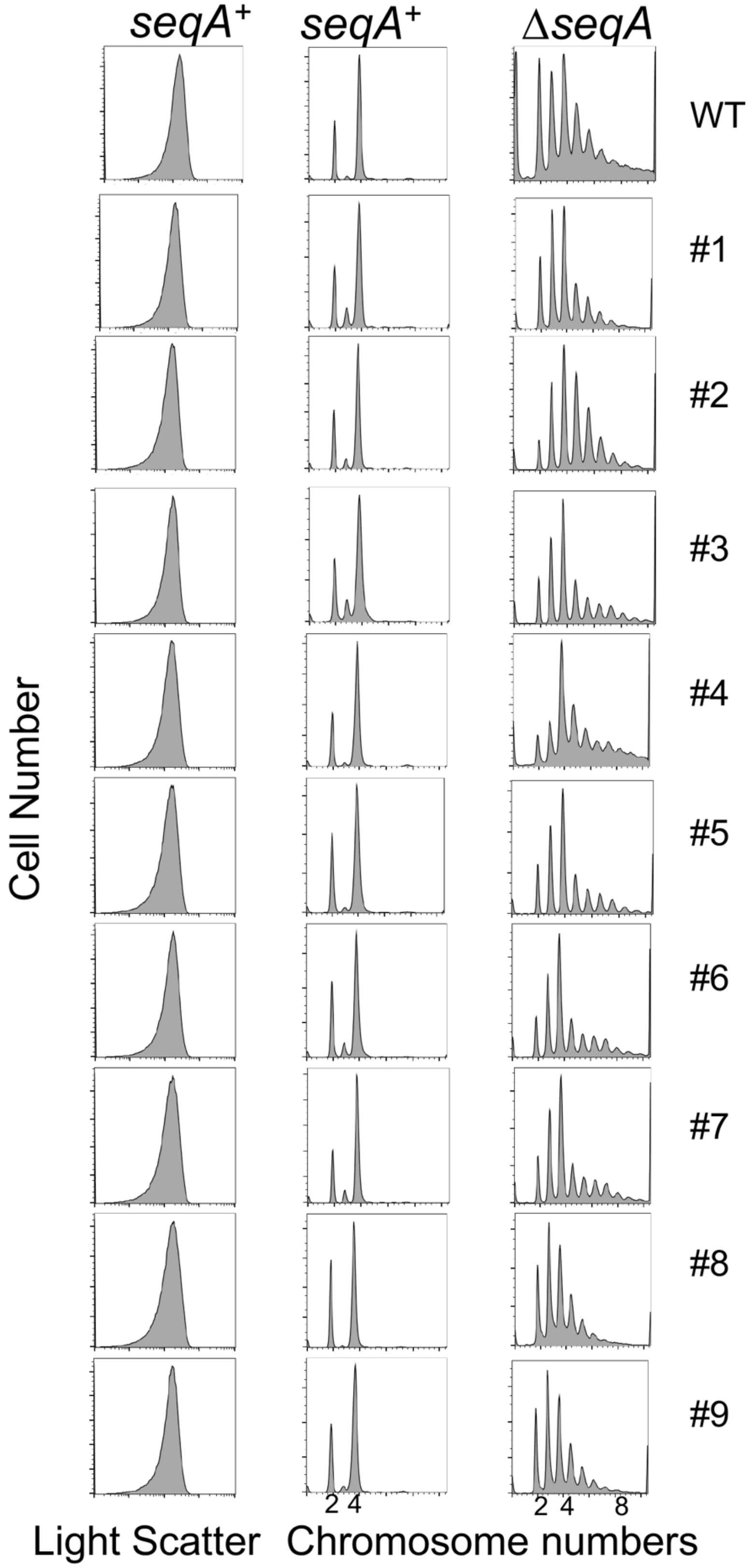

Figure B. DNA histogram of FRT marked isogenic *seqA*⁺ and Δ*seqA* strains of *oriC* mutants #1-9. These experiments were done as in Fig 3A except these cells did not have the R1 plasmids.

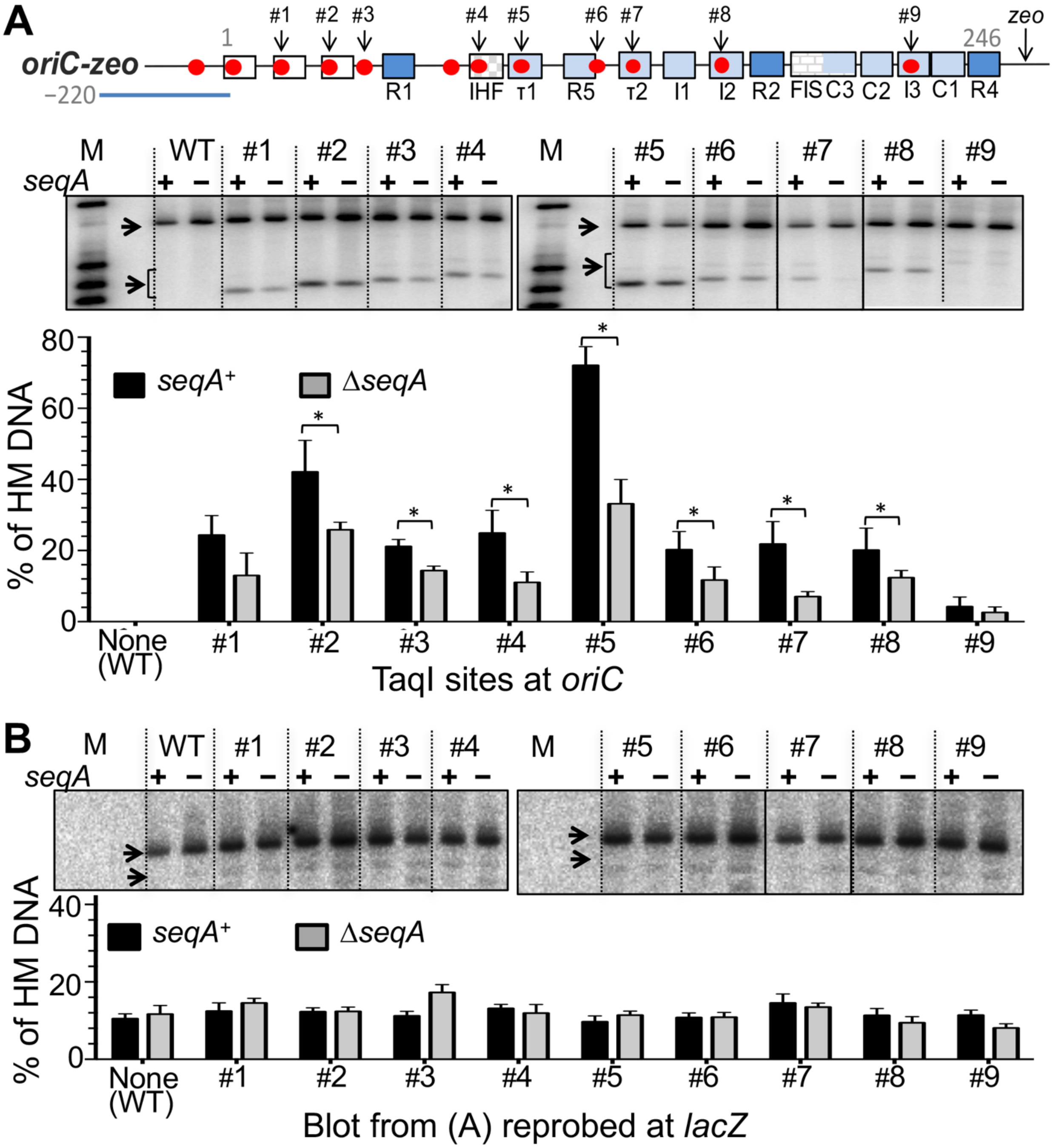

Figure C. HM DNA level at different GATC sites of *oriC* in *seqA*$^{+}$ and Δ*seqA* strains. The experiments in (A) and (B) were done similarly to those in Fig 2 A and B, except the strains were marked with *zeo* in place of the *FRT* site.

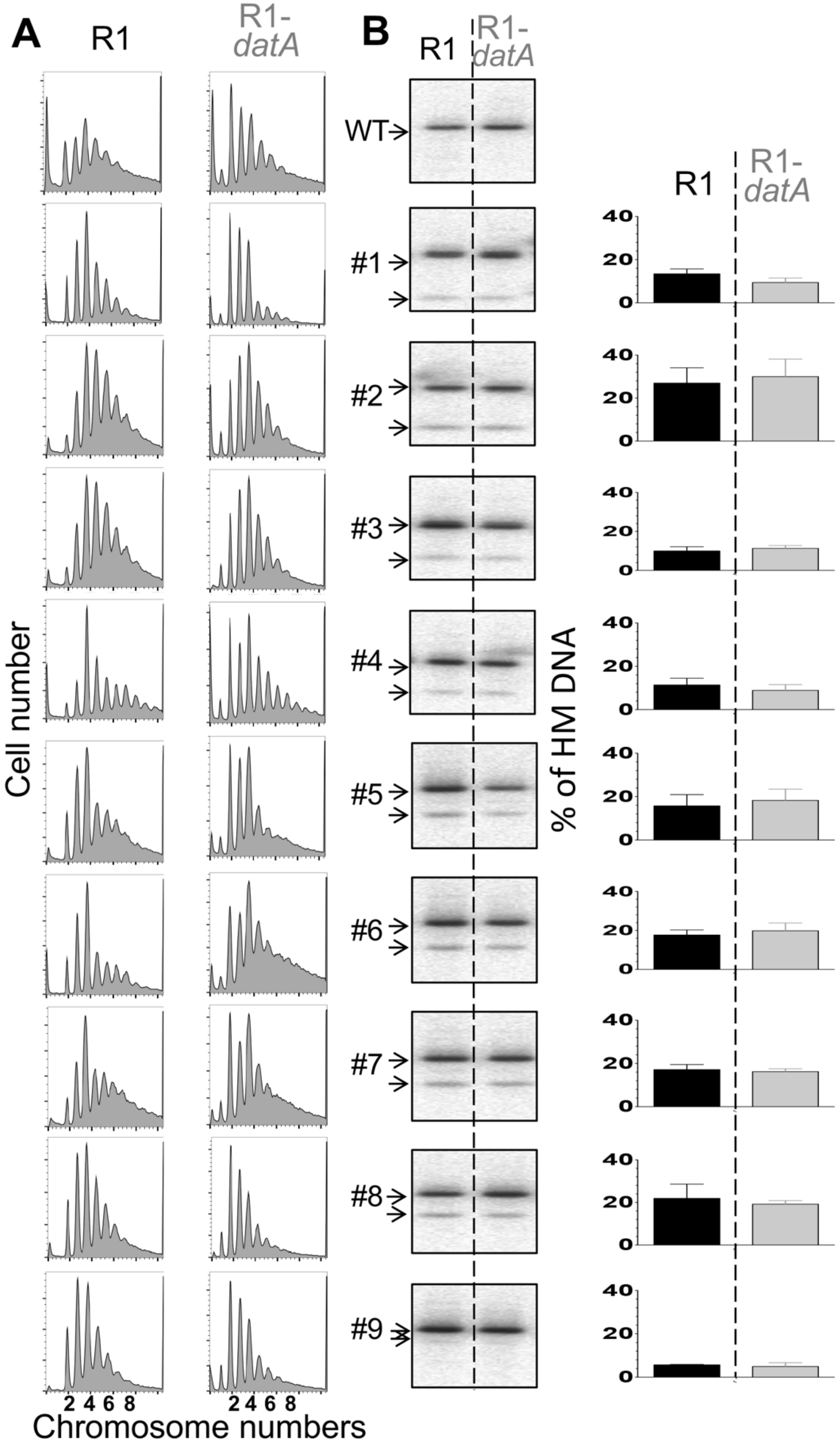

Figure D. Effect of DnaA titration on initiation synchrony and HM DNA level at *oriC*. The experiments in (A) and (B) were done similarly to those in Fig 3 A and B, except that the strains were Δ*seqA* derivatives of those used in Fig 3.

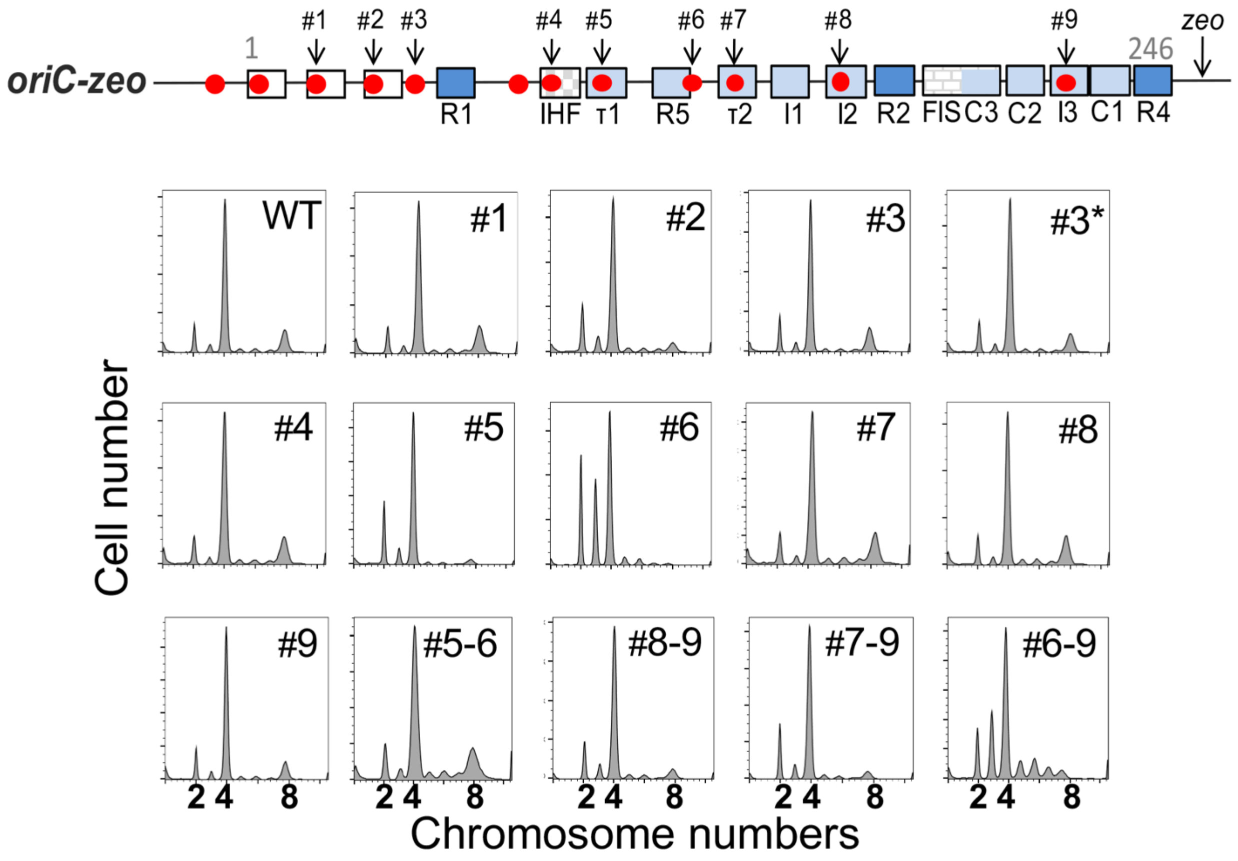

Figure E. Overcoming of initiation defect due to a mutation in #6 GATC in R5 by GATC mutations in #5 or #7-9 GATC of *oriC*. Chromosome contents of the GATC mutants were determined as in Fig 4A, except that CAA concentration was 0.5% instead of 0.1%. Note that initiation becomes more efficient in the double mutant #5-6 and in the quadruple mutant #6-9 compared to the single mutant #6.
